# Predictors of stress among a sample of Egyptian healthcare providers during the COVID-19 pandemic

**DOI:** 10.1186/s43045-021-00164-y

**Published:** 2021-11-17

**Authors:** Nermin Mahmoud Shaker, Noha Sabry, Muhammad Abdullatif Alkasaby, Menan Rabie

**Affiliations:** 1 Department of Neurology and Psychiatry, Faculty of Medicine, Ain Shams University, Cairo, Egypt; 2 Present Address: General Secretariat of Mental Health and Addiction Treatment, Ministry of Health and Population, Cairo, Egypt; 3 Department of Psychiatry, Faculty of Medicine, Cairo University, Cairo, Egypt

**Keywords:** COVID-19, Mental health, Healthcare workers, Perceived stress, Emergency response, Egypt

## Abstract

**Background:**

The COVID-19 pandemic had a tremendous effect on people’s mental health. Healthcare workers were on the front lines in response to this crisis; therefore, they were among the most affected by the pandemic. The study aims to assess the stress perceived by healthcare workers and possible factors contributing to it, hoping that more efforts will be exerted to support the well-being of healthcare workers during public health emergencies. A cross-sectional study using an online survey was conducted. Data were collected from 118 healthcare professionals working with COVID-19 patients during the peak of the first wave of the COVID-19 pandemic.

**Results:**

Most of the respondents (75.2%) scored much higher stress levels than average, and 19.5% had slightly higher levels of stress. Most respondents were afraid of infecting their family and close ones (77.1 %), and about half of the respondents were afraid of getting infected (47.5%). Regression analysis revealed that the only significant independent variable predicting developing higher stress levels among the participants was assigning them to tasks outside their specialty.

**Conclusions:**

Healthcare workers are at high risk of developing mental health problems during public health emergencies. Their well-being is essential for the quality of services they provide. More efforts are needed to ensure the well-being of healthcare workers and to prepare them for such emergencies. Preparing healthcare workers before redeployment through training and providing PPEs will help to reduce the negative impact of the COVID-19 pandemic on their physical and mental health.

## Background

On 11 March 2021, and after the spread of the novel coronavirus in 114 countries, the World Health Organization (WHO) has declared COVID-19 as a global pandemic [1]. Since then, the burden of this pandemic on mental health and the demand for mental health services has been increasing day after day. Studies suggest several factors associated with the COVID-19 pandemic that led to increased mental health problems among the population. Restrictions imposed on citizens to reduce the transmission of infections, as social distancing, and lockdown, had a negative effect on people’s mental health. Other factors include lack of information about the novel virus and the possibility of treatment, exposure to news and social media, and fear of infection. Economic factors as unemployment and low income are also contributors to the distress caused by the pandemic [2–4].

Health systems and health workers worldwide have been heavily affected by the COVID-19 pandemic as they were on the frontline in the face of the pandemic. Globally, about 10 % of COVID-19 infections are among healthcare workers [5]. On 2 September 2020, the Pan American Health Organization (PAHO) reported that about 570,00 health workers had been infected, and 2500 had lost their lives to COVID-19 in the region of the Americas [6].

In addition to its physical impacts, the pandemic has hugely impacted healthcare workers’ psychological and social well-being. In the amid of this crisis, medical staff have had to work under stress for long hours without rest putting themselves at risk of developing psychological adversities. In some studies, the prevalence of mental health problems such as stress, anxiety, depression, insomnia, and fear among healthcare workers has reached over 90%. This percentage was high among healthcare workers in direct contact with COVID-19 suspect cases and in countries most affected by the pandemic [7, 8]. Studies suggest some possible risk factors for the adverse psychological effects of the COVID-19 pandemic on healthcare workers. The most common factors were exposure to COVID-19 patients and worrying about being infected and infecting their family members and loved ones [8].

In Egypt, the COVID-19 pandemic had a huge impact on the country's healthcare system and health workers. More than 605 doctors have lost their lives to COVI-19 so far [9]. In addition, a large proportion of healthcare workers suffered from mental health problems, including stress, anxiety, and depression. The proportion of mental health problems were higher among female healthcare workers, young-age staff with less experience, and medical staff working in intensive care units, fever, and quarantine hospitals [10–13].

## Objectives

With the beginning of the declaration of the COVID-19 as a global pandemic, the General Secretariat of Mental Health and Addiction Treatment (GSMHAT) in Egypt has established the National Mental Health and Psycho-Social Support Coordination Group. This coordination group includes representatives from the government, non-governmental organizations, academic institutions, and the World Health Organization (WHO). This group aims to coordinate the Mental Health and Psycho-Social Support (MHPSS) activities during the pandemic. Several task forces were created under the coordination group, and the research task force was one of those task forces.

The current study was a part of the efforts made to support health workers on the frontlines. We aimed at screening for perceived stress and its predictors among a sample of healthcare providers during the COVID-19 crisis. This can help us to gain more understanding of the problem and develop interventions and programs targeting the psychological well-being and resilience of healthcare workers. A specific hotline was dedicated to those who had high levels of stress and needed support. Psychological support for healthcare workers is crucial not only for the staff themselves but also for the quality of the services they are providing and the safety of their patients.

## Methods

### Study design, setting, and participants

A cross-sectional study was conducted using an anonymous online survey to collect data between 21 May and 7 July 2020. The time of disseminating the survey coincides with the peak of the first COVID-19 wave, which was the most stressful period facing healthcare providers because of the increased workload and the lack of knowledge and experience in dealing with confirmed and suspected cases of the COVID-19 virus. The survey link was sent directly to the healthcare workers in the facilities dedicated to treating COVID-19 patients. The facilities included in our sample were among the first isolation hospitals opened at the beginning of the first COVID-19 wave in Cairo, Helwan, Aswan, Marsa Matrooh, Luxor, Alexandria-Asyout-Al-Qalubia, Al-Menia, Al-Behera, and Al-Gharbia. They included both governmental and university isolation hospitals. In addition, one fever hospital (Hommiat Aswan) and one chest hospital (Al-Maamora) were included. Those hospitals were chosen as the psychosocial support offered by GSMHAT was available in them.

The sample included doctors, nurses, and lab technicians. Convenient sampling was used, and 118 responses were received from participants during the period of the study.

### Data collection tools

A Google form questionnaire was created to collect the data from the participants. The designed form included informed consent, socio-demographic data of the participants, the Arabic version of the Perceived Stress Scale (PSS-10), and questions about possible predictors of stress during their practice. The informed consent explained the aim of the study, the anonymity, and the confidentiality of the survey. It also explained that the data would be used for research and improving the participants’ work conditions, then it was followed by a request for agreement to participate. The socio-demographic data collected included age, sex, place of work, and specialty.

The Arabic version of the Perceived Stress Scale (PSS-10) [14, 15] is a widely used self-reported scale for measuring the perception of stress. The scale consists of 10 items that measure how some life events are perceived as stressful. It asks about thoughts and feelings during the last month. Participants determine the frequency of experiencing specific items on a Likert scale from 0 to 4, where 0 means never, and 4 means very often. The score was obtained by reversing the score of the four positively stated items (item nos.4, 5, 7, and 8) then summing the score of all items. A score less than 16 is considered average, from 16-20 is slightly higher than average stress, and above 20 is considered much higher than average perceived stress. High levels of stress are a risk factor to different health problems, which necessitates intervening to reduce stress levels, such as changing lifestyle. The participants were encouraged to follow the instructions of the World Health Organization (WHO) and the General Secretariat of Mental Health (GSMHAT) for healthcare workers to alleviate stress during the pandemic if they are mild to moderately distressed (PSS score below 20). For example, they were encouraged to take time to rest and relax at work or between work shifts. Keeping in touch with family and friends, talking about their thoughts and feelings can also be beneficial. They were also encouraged to maintain their daily routine as much as possible (meals and sleep) and to do other activities as exercising and reading. Lastly, they were advised to avoid unhealthy coping methods as using tobacco, alcohol, or drugs. Those who were severely stressed (PSS score above 20) were encouraged to ask for specialized psychological support service through the hotline dedicated to the psychological support of medical staff offered by the GSMHAT.

Questions about the possible predictors of stress included lack of a clear protocol for dealing with positive cases or suspected or undiagnosed cases, unavailability of self-protective clothing or insufficient training on wearing and taking them off, stress due to dealing with critical situations, stress due to increased workload or being assigned to new tasks not related to their specialty and experiences, fear of becoming infected with the virus or, fear of transmitting the virus to their families, in addition to mentioning other reasons.

### Statistical analysis

Data were analyzed using the Statistical Package for the Social Sciences version 21 (SPSS 21). Mean and standard deviation were used for describing the continuous data, while number and percentage described the categorical data. Comparisons between groups of categorical data were made using the chi-square test. Multivariate stepwise linear regression analysis was performed for detecting predictors of stress among the participants.

## Results

A total number of 118 responses were received. 59.5% of the respondents were males (*n* = 69), while females were representing 40.5% of the respondents (*n* = 47). The majority of the participants (53.2%, *n* = 59) were aged between 18 and 34 years, while 45.0% (*n* = 50) were between 35 and 54 years old. Only 1.8% (*n* = 2) were above the age of 54 years old. Physicians represented 89.9 % of the respondents, while nursing staff represented only 9.1 %. 38.2% of the physicians were specialists, 31.6% were residents, 16.1% were consultants, and 3.4% were intern doctors. Their specialty included anesthesia, intensive care, emergency, medicine, chest, tropical, surgery, microbiology, and clinical pathology. 68.6% were working in general hospitals, 19.5% were working in health centers, and 7.6% were working in university hospitals.

Regarding the Perceived stress scale, most of the respondents (75.2%) scored much higher stress levels than average, and 19.5% had slightly higher levels of stress. Table 1 reports the socio-demographics of the sample participants compared to their level of stress. There was a significant association between the age of participants and the level of stress perceived.

**Table 1:** Socio-demographics of the study participants compared to their level of stress

Demographics	Perceived stress						*χ*2	***P*** value
	Average		Slightly higher		Much higher			
	***N***	%	***N***	%	***N***	%		
**Sex**								
Male (*N* = 69)	5	83.3	14	63.6	50	59.5	1.39	0.499
Female (*N* = 47)	1	16.7	8	36.4	34	40.5		
**Age group**								
18–34 (*N* = 59)	2	33.3	12	54.5	45	45.2	9.56	0.049
35–54 (*N* = 50)	4	66.7	8	36.4	38	54.8		
55–65 (*N* = 2)	0	0	2	9.1	0	0		
**Profession**								
Nurse (*N* = 10)	0	0	1	5	9	11.1	6.59	0.582
Intern doctor (*N* = 4)	0	0	1	5	3	3.7		
Resident (*N* = 35)	3	60	6	30	23	28.4		
Specialist (*N* = 42)	0	0	9	35	32	39.5		
Consultant (*N* = 19)	2	40	3	15	14	17.3		
**Workplace**								
University hospital (*N* = 9)	0	0	0	0	8	9.8	3.0	0.559
General hospital (*N* = 81)	5	83.3	16	76.2	57	69.5		
Health center (*N* = 23)	1	16.7	5	23.8	17	20.7		

**Fig. 1:**
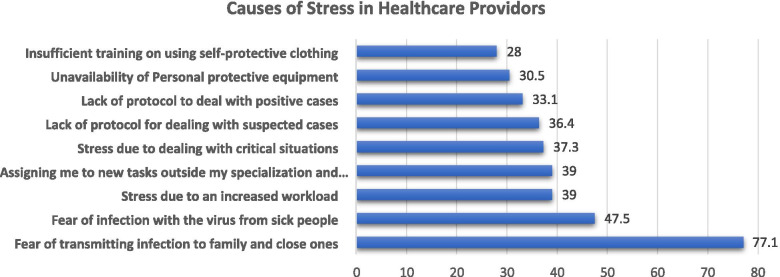
Causes of perceived stress. This figure shows the possible causes of stress among healthcare workers

Regarding the causes of stress perceived by healthcare workers, most respondents were afraid of transmitting the COVID-19 infection to their families and those close to them (77.1 %). In comparison, about half of the respondents were afraid to get infected (47.5%) (Fig. 1). Stepwise linear regression analysis revealed that the only significant independent variable predicting higher stress levels among the participants was assigning them to new tasks outside their specialization and experiences (Table 2).

**Table 2:** Stepwise linear regression analysis showing predictors of stress among the participants

Predictors of stress	Beta	***t***	sig
Assigning me to new tasks outside my specialization and experiences	0.267	2.92	**0.004**
The lack of a clear protocol for how to deal with positive cases	0.04	0.41	0.68
The lack of a clear protocol for how to deal with suspected or undiagnosed cases	0.08	0.90	0.37
Insufficient training on how to wear and take off self-protective clothing	0.05	0.53	0.60
Unavailability of personal protective equipment	− 0.03	− 0.03	0.76
Stress due to dealing with critical situations	− 0.04	− 0.45	0.65
Stress due to an increased workload	− 0.02	− 0.22	0.83
Fear of infection with the virus from sick people	0.04	0.44	0.66
Fear of transmitting the infection to my family and those close to me	− 0.02	− 0.21	0.84

## Discussion

The COVID-19 pandemic has affected the lives of billions and disrupted health systems all over the world. Besides its effect on physical health, the pandemic also has a huge impact on people’s mental health. The rate of mental health problems such as depression, anxiety, distress, and insomnia has tremendously increased during the pandemic. Some of the factors contributing to the increase in mental health problems during the pandemic include fear of being infected or losing a family member or close ones. Also, exposure to too much information, which can be wrong and misleading, was associated with an increased prevalence of mental health problems [16]. Governments all over the world have implemented preventive measures to reduce the spread of COVID-19 infections. Those measures have disrupted the social support networks that may help mitigate the impact of the pandemic on mental health.

Health workers were among those who were mostly affected by the pandemic. Thousands of health workers lost their lives to the pandemic, and hundreds of thousands got infected. The Egyptian medical syndicate has announced the death of 605 physicians from COVI-19 infection. Our study included 118 persons of the medical staff working in quarantine hospitals. Most of the respondents (75.2%) have shown a much higher level of stress, the median score of the perceived stress scale (PSS) was 24, and the interquartile range (IQR) was 20–27. Similar results were reported in a study by Rossi et al. [17], where the median was 24 and IQR 18–29. Comparing COVID-19 to other outbreaks, the percentages of healthcare workers who reported high stress levels ranged from 18 to 80% during the SARS outbreak [18].

In contrast to some studies which reported that female sex and young age are associated with increased mental health problems, our study shows no statically significant difference between sexes. Still, there was a statistically significant difference between age groups. Younger health workers were more prone to stress. This might be due to the lack of clinical experience in dealing with such stressful situations [10, 11, 17]. Regarding the predictors of stress among medical staff, the fear of transmitting the infection to family members and the fear of being infected were among the most stressful factors chosen by respondents (77.1% and 47.5% respectively). Those two factors were reported by several studies [8]. Additionally, increased workload, dealing with positive and suspected COVID-19 cases, unavailability of personal protective equipment (PPE), and the lack of training on their use were among the concerns reported by our study. Other concerns reported by healthcare workers include being unable to go home for a long time, lack of professional knowledge, and the death of their patients [19, 20]. It is important to target those risk factors through different interventions to mitigate the impact of the pandemic on healthcare workers’ mental health.

Redeployment of medical staff to intensive care units and other departments with greater demand was a common practice during the pandemic. The deployment has also included mental health professionals, which in turn added more burden on the mental health services provided [21]. In this study, assigning healthcare workers to new tasks outside their area of expertise was the only significant independent variable predicting developing higher stress levels among the participants. Supporting the redeployed healthcare workers through training and providing the supplies they need may help relieve the stress they are exposed to [22].

Healthcare workers are at high risk of developing mental health problems during emergencies. Their well-being is crucial for the quality of the service they provide. As a part of this study, respondents suffering from mild forms of stress were given some advice on healthy ways to cope with stress. Those suffering from high levels of stress were referred to a helpline operated by mental health specialists and dedicated to supporting medical staff. In addition, hundreds of healthcare workers in Egypt were trained on recognizing signs and symptoms of stress, healthy coping methods, and when to ask for help. Other forms of support include providing medical staff with the knowledge and supplies needed to perform their job properly [23].

## Limitations

Research in the emergencies context is challenging. Healthcare workers in emergency settings have priorities other than research. It was hard to reach out to the study population and take a large representative sample from all healthcare workers due to several challenges, including the restrictions imposed due to the pandemic. That is why we collected data using an online survey. Although participants with high levels of stress were given some advice regarding the healthy ways of coping with stress, and some were recommended to call the helpline, it was hard to follow them up due to the cross-sectional nature of the study.

## Conclusions

Healthcare workers are on the frontlines in response to the COVID-19 pandemic, and they are at a high risk of developing one or more mental disorders. Supporting healthcare workers, by all means, is essential. Redeploying medical staff to work with COVID-19 patients is a stressful event. To mitigate the pandemic’s adverse psychological and physical effects on healthcare workers, they should be provided with the equipment and training required to do their job effectively before redeployment.

## Data Availability

The datasets used and/or analyzed during the current study are available from the corresponding author on reasonable request.

## Abbreviations

GSMHAT: the General Secretariat of Mental Health and Addiction Treatment
MHPSS: Mental Health and Psycho-Social Support
PSS-10: the Perceived Stress Scale (10 items)
WHO: World Health Organization
